# Large-scale malaria survey in Cambodia: Novel insights on species distribution and risk factors

**DOI:** 10.1186/1475-2875-6-37

**Published:** 2007-03-27

**Authors:** Sandra Incardona, Sirenda Vong, Lim Chiv, Pharath Lim, Sina Nhem, Rithy Sem, Nimol Khim, Socheat Doung, Odile Mercereau-Puijalon, Thierry Fandeur

**Affiliations:** 1 Laboratory of Molecular Epidemiology, Institut Pasteur du Cambodge, Phnom Penh, Cambodia; 2 Epidemiology and Public Health Unit, Institut Pasteur du Cambodge, Phnom Penh, Cambodia; 3 National Centre for Parasitology, Entomology and Malaria Control, Phnom Penh, Cambodia; 4 Unité d'Immunologie Moléculaire des Parasites, Institut Pasteur, 28 rue du Dr Roux, 75724 PARIS cedex 15, France

## Abstract

**Background:**

In Cambodia, estimates of the malaria burden rely on a public health information system that does not record cases occurring among remote populations, neither malaria cases treated in the private sector nor asymptomatic carriers. A global estimate of the current malaria situation and associated risk factors is, therefore, still lacking.

**Methods:**

A large cross-sectional survey was carried out in three areas of multidrug resistant malaria in Cambodia, enrolling 11,652 individuals. Fever and splenomegaly were recorded. Malaria prevalence, parasite densities and spatial distribution of infection were determined to identify parasitological profiles and the associated risk factors useful for improving malaria control programmes in the country.

**Results:**

Malaria prevalence was 3.0%, 7.0% and 12.3% in Sampovloun, Koh Kong and Preah Vihear areas. Prevalences and *Plasmodium *species were heterogeneously distributed, with higher *Plasmodium vivax *rates in areas of low transmission. Malaria-attributable fevers accounted only for 10–33% of malaria cases, and 23–33% of parasite carriers were febrile. Multivariate multilevel regression analysis identified adults and males, mostly involved in forest activities, as high risk groups in Sampovloun, with additional risks for children in forest-fringe villages in the other areas along with an increased risk with distance from health facilities.

**Conclusion:**

These observations point to a more complex malaria situation than suspected from official reports. A large asymptomatic reservoir was observed. The rates of *P. vivax *infections were higher than recorded in several areas. In remote areas, malaria prevalence was high. This indicates that additional health facilities should be implemented in areas at higher risk, such as remote rural and forested parts of the country, which are not adequately served by health services. Precise malaria risk mapping all over the country is needed to assess the extensive geographical heterogeneity of malaria endemicity and risk populations, so that current malaria control measures can be reinforced accordingly.

## Background

Cambodia has long been a region of endemic malaria. After much inconsistencies and political turmoil, the country has now established an efficient malaria control programme and has recently been cited as one of the countries most efficiently fighting against malaria [1]. Between 1997 and 2005, the number of malaria cases decreased by 57 %, thanks to the complementary control strategies implemented during the last decade by the National Centre for Parasitology, Entomology and Malaria Control (previously 'Centre National de Malariologie' CNM) [2-5]. Nevertheless, rates of mortality and morbidity are still at unacceptable levels in some areas, justifying continued efforts to combat the disease [6]. About 74,185 malaria cases were reported by CNM in 2005, of which 49,436 were confirmed by laboratory diagnosis The clinically most relevant species, *Plasmodium falciparum*, accounted for 80% of infections [3].

Current estimates of the malaria burden in Cambodia rely on the data collected by the public health information system (HIS) that does not record malaria cases occurring among remote populations. These data include symptomatic patients consulting in public sector health facilities [2-4,7], whereas malaria cases treated by traditional healers or private practitioners remain unrecorded. Although clinical malaria incidence has decreased, the extent and distribution of asymptomatic carriage that significantly contributes to transmission is, therefore, missed by the present information system. Furthermore, only a few studies on malaria prevalence and parasite species distribution have been published so far in Cambodia. These were limited to ethnic comparisons, entomological studies, surveys of refugees or migrant workers, and thus their relevance for the actual prevailing malaria situation is unclear [8-11]. Two independent studies have shown that the private health sector plays a large role in malaria case management, especially in remote areas, with only 18–19% of individuals seeking their first treatment at the nearest public health facility [12,13]. The global malaria situation and associated risk factors are still under-documented in Cambodia.

To gain a better insight into the malaria situation at the province level, malaria parasitologic profiles and spatial distribution of infections in populations living along the Thai border, in three areas of multidrug resistant malaria were determined [5,14,15]. Detailed parasitologic data were used to derive baseline information for planning and updating malaria control programmes in these areas.

## Study subjects and methods

### Study areas

**Figure 1:**
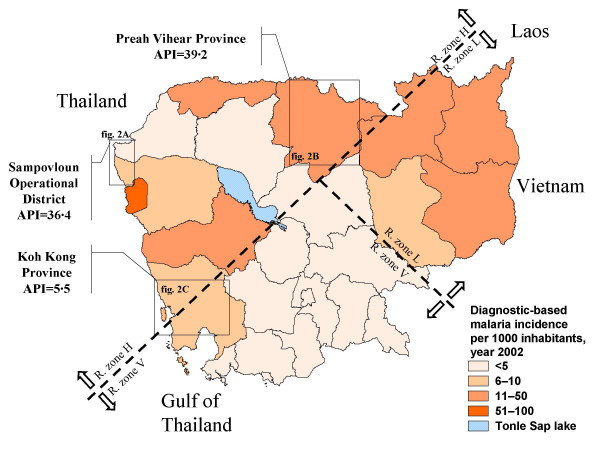
**Reported malaria cases and *P. falciparum *drug-resistance areas in Cambodia, year 2002**. The annual parasite incidence (API) per 1,000 inhabitants, confirmed by blood-slide examination or rapid diagnosis test, is shown for each province of Cambodia (source: National Malaria Center, 2002). The country is also divided in areas of low (L), variable (V) and high (H) resistance of *P. falciparum *to chloroquine. The three surveyed regions and their respective annual parasite incidences in 2002 are indicated (detailed maps in Figure 2).

The national HIS indicates a high malaria incidence in the border provinces of Cambodia (Figure 1) where the major malaria vectors *Anopheles dirus*, *Anopheles minimus *and *Anopheles maculatus *are widespread in forest-covered hills and mountains [5,7,16,17]. The study focused on three regions bordering Thailand: the Preah Vihear province in the North; the Sampovloun operational district (Battambang province) in the North-west; and the Koh Kong province in the South-west. In 2002, the recorded annual parasite incidence per 1,000 inhabitants (API) in Sampovloun, Koh Kong and Preah Vihear was of 36.4, 5.5 and 39.2, respectively, whereas the national average was of 3.6 (Dr Tol Bunkea, CNM, unpublished results) [3]. These provinces are located in an area of *P. falciparum *multidrug resistance [5,14,15]. No data were available for Koh Kong itself, but high levels of *in vivo *and *in vitro *drug resistance were reported in neighbouring provinces of Pursat and Kompong Speu, which border Koh Kong in the North and the East, respectively.

Malaria transmission occurs mostly during the rainy season, with substantial regional and year-to-year variations. The survey periods, namely : November/December 2001 in Sampovloun, March 2002 in Koh Kong and August/September 2003 in Preah Vihear, were chosen according to previous CNM records (Dr Tol Bunkea, CNM, unpublished results) [3] in order to coincide with the respective transmission seasons.

### Study design and subjects

The sample size to be screened was calculated to yield a 95 % CI and a precision level of 1% using Sampsize software [18]. Based on the total population size in these provinces, estimated to be 225095 from the last census, and after excluding urban centers and places where no malaria transmission occurs, a sample size of 9211 persons was calculated. Villages were randomly selected from the 1998 nationwide census village list. Some logistically inaccessible villages were replaced by the closest accessible village. In Sampovloun, some villages were divided between the villages themselves and the so-called "chamkar" (farms), which were considered as one village entity. In numerous places the composition and location of households was highly heterogeneously distributed precluding an appropriate randomization of households which furthermore would not have yielded the aimed sample size. A global sampling method aiming at coverage of all inhabitants was used. Village chiefs were informed about the purpose and date of the visit to ensure maximum participation of the villagers. All villagers present on the scheduled day of the survey were enrolled under the supervision of the village Chiefs and malaria control agents from the Ministry of Health. For each participant, informed consent was obtained, an identification code was issued, and age, gender, bednet use were recorded. The occupation of the individuals was recorded and classified in three categories: 'inside village' ('child', 'school', 'staying at home'), 'outside village' ('farmer', 'military'), and 'other' ('fisher', 'vendor', 'NGO agent', 'unknown'). The axillary temperature was measured and splenomegaly was determined using the Hackett classification (grade 1–5). For individuals with fever or other malaria-related symptoms, a rapid diagnosis test was carried out (Paracheck Pf, Orchid, India or OptiMAL-IT, Diamed, Switzerland). Individuals testing positive were treated according to national guidelines (artesunate + mefloquine for *Plasmodium falciparum *infections, chloroquine for *Plasmodium vivax *infections). Fingerprick blood was collected from all individuals for both thin and thick smears. The study was approved by the Cambodian National Ethics Committee.

### Microscopy and case definitions

Thin smears were fixed with methanol, and slides were stained with 3% Giemsa for 30 min. The slides were examined by experienced microscopists at the CNM. At least 100 thick film fields at 1,000× magnification were examined before a slide was considered negative. Parasite species and stages were confirmed on the thin film. Densities were scored as follows: 1+ for 1–10 parasites per 100 thick film fields; 2+ for 11–100 parasites per 100 fields; 3+ for 1–10 parasites per single field; 4+ for 11–100 parasites per single field; 5+ for > 100 parasites per single field (WHO, Basic Malaria Microscopy, Geneva, Switzerland)

Malaria infection was defined as the presence of *Plasmodium *parasites on microscopy slides. Malaria-attributable fever cases and symptomatic malaria cases were defined as individuals with fever and a positive microscopy result, irrespective of the parasite density [19].

Splenomegaly was either positive (Hackett grade 2–5) or negative (Hackett grade 1). Fever was defined as an axillary temperature ≥ 37.5°C.

### Geographical positioning and analysis

The coordinates of the survey villages were taken from the 1998 census database (National Institute of Statistics, Cambodian Ministry of Planning). The villages not listed in the database were recorded on site using a Global Positioning System. The coordinates of the government health facilities were taken from the 2003 Administrative Health Facility Mapping database (National Institute of Statistics). Roads, water and forest data were from the 2003 Cambodia Reconnaissance Survey Digital Data of the Cambodian Ministry of Public Works and Transportation and the Japan International Cooperation Agency (JICA), which derived from various sources (maps, aerial and satellite photographs, field verifications) dating from 1992–1996 in Koh Kong, and from 1994–2002 in Preah Vihear and Sampovloun.

The ArcGIS v9.0 software (ESRI Systems, Redland, CA, USA) was used to measure the distances between villages and i) the nearest public health care service, and ii) the nearest forest potentially harboring malaria vectors, using Euclidian distance measurements, the category "evergreen broad leafed forest" was considered most likely associated with a high *A. dirus *and *A. minimus *abundance, in agreement with experience and data of the Entomology Department of the CNM (Dr Tho Sochantha, CNM, unpublished results) [3].

### Statistical analysis

An anonymous database was created and double-checked. Stata v8.0 was used for all statistical analyses. Association of the individual's characteristics and the village's distance-to-forest and distance-to-health-facility covariates with malaria prevalence was analyzed by a generalized linear mixed model with a logistic regression function (Stata command 'gllamm', Generalized Linear Latent and Mixed Model). Variables with a *p *value < 0.05 in the bivariate analysis were retained in the model. Variables with correlation coefficients > 0.70, indicating co-linearity, were not included together in the model. Interaction between relevant risk factors was assessed by comparing the models with and without the interaction factor using the log-likelihood ratio test and a significant *p *value of < 0.05.

## Results

### Study population

About 11,652 individuals from 88 villages accounting for 2%-8% of the targeted populations in the three areas were tested for malaria (Table 1). Most of the participants were between 5 and 39 years old. The male/female ratio was lower in the adult population aged ≥ 15 years than in < 15 year old children (0.56 versus 0.97). Most individuals had activities within the village, although a large number (39–43%) regularly worked outside the village. Bednets were used by most individuals, particularly in Sampovloun and Koh Kong areas (98% for both).

**Table 1:** Characteristics of study population and univariate analysis of malaria risk factors

	**Sampovloun**					**Koh Kong**					**Preah Vihear**				
	**Surveyed**		***P. spp*. positive**			**Surveyed**		***P. spp*. positive**			**Surveyed**		***P. spp*. positive**		
	No	%	No	%	p	No	%	No	%	p	No	%	No	%	p
No of villages	27					38					23				
No of individuals	4074		123	3.0		2624		184	7.0		4954		610	12.3	
															
Age															
< 5 years	363	8.9	4	1.1	< 0.001	229	8.7	7	3.1	< 0.001	922	18.6	112	12.2	0.002
5–14 years	1586	38.9	26	1.6		915	34.9	79	8.6		1748	35.3	242	13.8	
15–39 years	1434	35.2	76	5.3		966	36.8	78	8.1		1739	35.1	214	12.3	
≥ 40 years	691	17.0	17	2.5		514	19.6	20	3.9		545	11.0	42	7.7	
															
Gender															
female	2258	55.4	43	1.9	< 0.001	1513	57.7	83	5.5	< 0.001	2938	59.3	323	11.0	0.001
male	1816	44.6	80	4.4		1111	42.3	101	9.1		2016	40.7	287	14.2	
															
Occupation*															
inside village	2183	53.6	35	1.6	< 0.001	1216	46.3	81	6.7	0.023	2910	58.7	378	13.0	0.07
outside village	1764	43.3	88	5.0		1027	39.1	95	9.3		2015	40.7	227	11.3	
															
Bednet use															
Yes	3996	98.1	117	2.9	0.015	2569	97.9	180	7.0	0.939	4313	87.1	496	11.5	< 0.001
no	78	1.9	6	7.7		55	2.1	4	7.3		641	12.9	114	17.8	

* unclassified occupations were excluded

The age, gender, occupation and bed-net use distribution is shown for the surveyed population (representing 7.7%, 1.9% and 4.0% of the population covered by the public health system in Sampovloun, Koh Kong and Preah Vihear, respectively), and for the individuals testing positive for *Plasmodium *by microscopy. *P *values from a univariate analysis of malaria prevalence distribution in each area are also shown.

### Malariometric indices

Data on parasite prevalence, spleen enlargement and fever rates are presented in Table 2. *Plasmodium spp*. prevalence was 3.0%, 7.0% and 12.3% in Sampovloun, Koh Kong and Preah Vihear, respectively. *P. falciparum *was predominant in Preah Vihear and Koh Kong (75% and 65% of positive cases, respectively), whereas nearly half of the positive cases in Sampovloun were due to *P. vivax*. Mixed infections, mainly *P. falciparum *and *P. vivax*, were detected in 6–9% of positive slides. Eight *Plasmodium malariae *infections were identified during the survey, but *Plasmodium ovale *was not observed.

**Table 2:** Malaria indicators and morbidity by study area

	**Sampovloun**		**Koh Kong**		**Preah Vihear**	
	No	(%)	No	(%)	No	(%)
**Microscopy diagnosis**						
No of examined slides	4074		2624		4954	
						
*P. spp*. positive	123	(3.0)	184	(7.0)	610	(12.3)
						
single infections	115	(2.8)	167	(4.1)	573	(11.6)
*P falciparum*	59	(1.4)	120	(2.9)	455	(9.2)
*P vivax*	56	(1.4)	46	(1.1)	115	(2.3)
*P malariae*	0	(0.0)	1	(0.02)	3	(0.1)
						
mixed infections	8	(0.2)	17	(0.4)	37	(0.7)
*P falciparum/vivax*	8	(0.2)	14	(0.3)	36	(0.7)
*P falciparum/malariae*	0	(0.0)	3	(0.1)	1	(0.02)
						
**Gametocyte carriage**						
*P. falciparum*	6	(9.0)	20	(14.6)	53	(10.8)
*P. vivax*	7	(10.9)	2	(3.3)	0	(0.0)
						
Spleen rates*						
No of surveyed individuals	3451		2623		4954	
with splenomegaly	46	(1.3)	23	(0.9)	60	(1.2)
						
No of children 2–9 years old	839		746		1620	
with splenomegaly	10	(1.2)	11	(1.5)	35	(2.2)
						
No of *P. spp*. positive individuals	98		184		610	
with splenomegaly	9	(9.2)	11	(6.0)	36	(5.9)
						
Fever rates*						
No of surveyed individuals	3842		2623		4954	
No of individuals with fever	210		415		603	
malaria attributable fever cases†	35	(16.7)	42	(10.1)	199	(33.0)
						
No of *P. spp*. positive individuals	117		184		610	
symptomatic malaria cases†	35	(29.9)	42	(22.8)	199	(32.6)

* For some individuals, fever and splenomegaly data were not available.

† *P. spp*. positive individuals with fever

Results of microscopy diagnosis and clinical examination are shown for each surveyed area. Percentages of *Plasmodium *and subspecies infections are with respect to total numbers of examined slides, while rates of splenomegaly and fever are with respect to total numbers of surveyed individuals.

*P. falciparum *gametocyte carriage, ranging from 9 to 15% of the positive cases, did not differ significantly in the three survey areas. By contrast, *P. vivax *gametocytes were detected at lower rates and solely in Sampovloun and Koh Kong areas (Table 2). In Preah Vihear, *P. falciparum *gametocytes were more frequent in < 5 year old children than in older age groups (17.2% versus 9.4%, *p *= 0.033). Similarly, sexual forms were significantly most prevalent in samples with low grade parasite densities (15.4% versus 5.5%, *p *< 0.001).

Spleen rates were low among the surveyed population and in 2–9 year old children (1–2%), but were higher in parasite-positive individuals, reaching 6–9%. In Preah Vihear and Koh Kong areas, where malaria transmission is more marked, spleen enlargement was more frequent in children aged < 15 years than in individuals aged ≥ 15 years (2.0% versus 0.3%, *p *< 0.001 and 1.5% versus 0.4%, *p *= 0.003, respectively) whereas in Sampovloun, it was only observed in individuals aged ≥ 5 years.

### Morbidity and parasite density

In Preah Vihear, 33% of fever cases were associated with a *Plasmodium spp*. infection, versus 10% and 17% in Koh Kong and Sampovloun, respectively (Table 2). As a consequence, the positive predictive values of fever as a presumptive diagnosis criterion was very low (PPV of 16.7%, 10.1% and 33.0%, in Sampovloun, Koh Kong and Preah Vihear, respectively). Symptomatic malaria infections were observed in approximately one-quarter (Koh Kong) and one-third (Sampovloun, Preah Vihear) of parasite-positive individuals, and were significantly more frequent in men than in women for the three areas (37.4% versus 23.0%, *p *< 0.001). In Preah Vihear, *P. falciparum *parasite density was higher in < 5 year old children than in the older age groups (densities > 2+ being observed in 45.1% versus 28.8% of infections, *p *= 0.004). In all the three survey areas, fever rates increased with parasite densities, especially between densities < 3+ and ≥ 3+ for both *P. falciparum *(27.9% versus 45.1%, *p *< 0,001) and *P. vivax *(20.1% versus 63.9%, *p *< 0.001).

### Spatial distribution of prevalence

**Figure 2:**
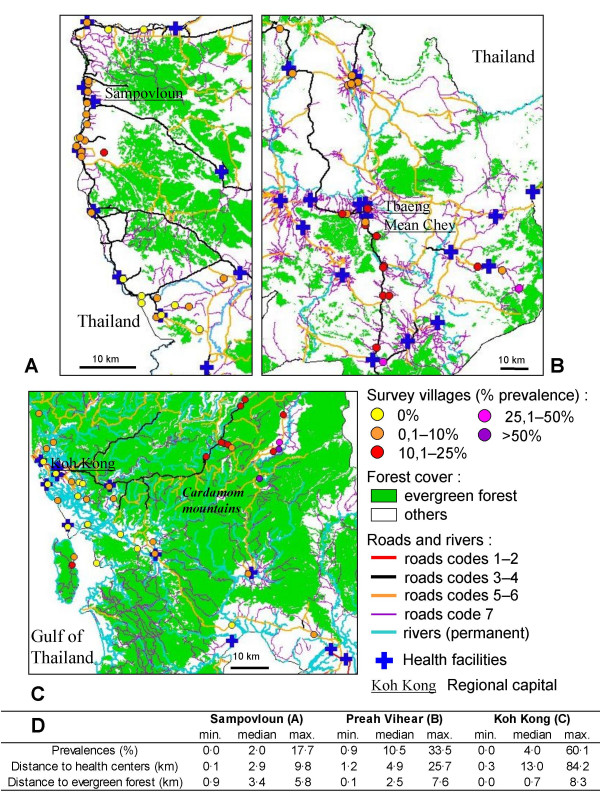
**Maps of the study areas Sampovloun (A), Preah Vihear (B) and Koh Kong (C)**. Maps 2A, B and C show the distribution of microscopy-based age/gender-standardized malaria prevalence in the surveyed villages, evergreen forest, roads according to their quality (codes 1–2: hard surface roads passable in all weather, codes 3–4: loose surface roads passable in all weather, codes 5–6: cart tracks and loose surface roads passable in dry weather only, code 7: footpaths), rivers, and positions of public health facilities. Figure 2D shows the median, minimum and maximum values of village-based age/gender-standardized *Plasmodium *prevalence, distances to public health facilities and distances to evergreen forest.

As visualized in Figure 2, villages displaying the highest malaria rates are clustered along roads or tracks penetrating into recently colonized forested areas not served by health services. Koh Kong displayed the greatest spatial heterogeneity, with village prevalence ranging from 0% in the coastal area up to 60.1% in the Cardamom mountains (Figure 2C). The highest prevalences of malaria infection were clustered in remote villages located about 60 km north-east of Koh Kong city, in a mountainous and forested area not covered by public heath centers. Notably, the villages most at malaria risk, with prevalence rates above 50%, are located in remote places, accessible by road during the dry season only. In Preah Vihear, forests and health facilities were more uniformly distributed, and prevalence varied from 0.9% to 33.5% (Figure 2B). In the north of this province, a cluster of villages with low prevalence (< 10%) coincided with a lack of evergreen forest in the surrounding areas. In the south, numerous villages with high prevalence have settled along the main road leading to the Thai border, which apparently constitutes a route along which malaria parasites do circulate and spread over the entire province. The narrowest difference was observed in Sampovloun, with uniformly low prevalence (≤ 8.5%), except in one village (17.7%) which actually corresponds to a forest camp ("chamkar"). The distance to the nearest health facility never exceeded 10 km, and the median distance to evergreen forest was the greatest of the three areas (Figure 2A). An effect of the distance from the villages to forests and to health facilities was observed in the three areas. The prevalence rate increased concomitantly with the distance to health center whereas an inverse relation was observed with the distance to forest fringe (Figure 2D).

### Risk factors

Gender, age, bednet use and occupation significantly influenced the risk of *Plasmodium spp*. infections, depending on the survey area (Table 1). The factor "occupation" was excluded from further analyses because of its strong co-linearity with age in all areas (R^2 ^= 0.68–0.73). Multivariate regression combining all individual risk factors covariates confirmed the significant effect of the distance-to-forest and distance-to-health-facility (Table 3). Males had an increased risk of *Plasmodium spp*. infection in the three areas, the highest risk (OR = 2.306) being observed in Sampovloun. The age group at highest risk of malaria differed in each area, being 15–39 years in Sampovloun, 5–39 years in Koh Kong, and 0–39 years in Preah Vihear. In Sampovloun, we found a significantly increased risk for males aged 15–39 years, due to an interaction between age and gender. A protective effect of bednets was detected in Preah Vihear. Due to the large coverage and regular use of bednets by population in other provinces, such an effect was not detected in Koh Kong and Sampovloum by the multivariate analysis, though it was readily established by the univariate analysis in Sampovloun (Table 1). An increased distance to forest tended to reduce the infection risk in Preah Vihear and in Koh Kong, whereas an increased distance to health facilities tended to increase the risk of malaria infection.

**Table 3:** Multivariate analysis of risk factors

***Plasmodium spp*.**	Sampovloun*			**Koh Kong**			**Preah Vihear**		
**Variables at individual level : (tested/reference)**									
	**OR**	**95% CI**	***p***	**OR**	**95% CI**	***p***	**OR**	**95% CI**	***p***
gender (male/female)	2.31	1.56–3.41	< 0.001	1.69	1.19–2.38	0.003	1.31	1.10–1.57	0.003
age (0–5 years/≥ 40 years)	0.4	0.13–1.23	0.109	1.53	0.59–3.95	0.377	1.41	0.96–2.07	0.084
age (5–14 years/> 40 years)	0.6	0.32–1.14	0.118	3.46	1.96–6.11	< 0.001	1.85	1.30–2.65	0.001
age (15–39 years/> 40 years)	1.97	1.14–3.41	0.016	2.68	1.53–4.67	0.001	1.63	1.14–2.33	0.008
bednet (used/not used)	0.65	0.26–1.65	0.364	1.79	0.54–5.95	0.341	0.6	0.46–0.77	< 0.001
**Variables at village level : (increasing distances)**									
	**OR**	**95% CI**	***p***	**OR**	**95% CI**	***p***	**OR**	**95% CI**	***p***
distance to forest	1.21	0.85–1.74	0.146	0.8	0.59–1.07	0.065	0.82	0.72–0.94	0.003
distance to health center	0.86	0.70–1.07	0.085	1.05	1.04–1.07	< 0.001	1.03	0.99–1.08	0.076

* Sex-age interaction was observed upon analysis with two ageclasses (tested/reference : age ≥ 15 years/< 15 years) sex × age: OR = 4.37, 95% CI = 1.82–10.47, p = 0.001, likelihood-ratio test A: LR = 11.08, p < 0.001									

***Plasmodium falciparum***	**Sampovloun**			**Koh Kong**			**Preah Vihear**		

**Variables at individual level : (tested/reference)**									
	**OR**	**95% CI**	***p***	**OR**	**95% CI**	***p***	**OR**	**95% CI**	***p***
gender (male/female)	2.75	1.61–4.73	< 0.001	1.67	1.13–2.46	0.01	1.43	1.17–1.75	< 0.001
age (0–5 years/> 40 years)	0.3	0.07–1.40	0.127	1.03	0.32–3.31	0.965	1.19	0.78–1.82	0.425
age (5–14 years/> 40 years)	0.3	0.12–0.75	0.01	2.86	1.52–5.38	0.001	1.7	1.16–2.51	0.007
age (15–39 years/> 40 years)	1.77	0.89–3.50	0.101	2.51	1.36–4.64	0.003	1.48	1.00–2.18	0.049
bednet (used/not used)	0.37	0.13–1.05	0.06	1.79	0.47–6.76	0.393	0.56	0.42–0.73	< 0.001
**Variables at village level : (increasing distances)**									
	**OR**	**95% CI**	***p***	**OR**	**95% CI**	***p***	**OR**	**95% CI**	***p***
distance to forest	1.2	0.80–1.81	0.191	0.85	0.63–1.15	0.15	0.82	0.68–0.99	0.018
distance to health center	0.87	0.69–1.10	0.121	1.05	1.03–1.06	< 0.001	1.05	0.99–1.12	0.049

***Plasmodium vivax***	**Sampovloun**			**Koh Kong**			**Preah Vihear**		

**Variables at individual level : (tested/reference)**									
	**OR**	**95% CI**	**p**	**OR**	**95% CI**	**p**	**OR**	**95% CI**	**p**
gender (male/female)	1.94	1.15–3.28	0.013	1.29	0.75–2.21	0.357	1.06	0.76–1.48	0.725
age (0–5 years/> 40 years)	0.91	0.22–3.74	0.899	2.13	0.49–9.27	0.312	2.84	1.24–6.49	0.014
age (5–14 years/> 40 years)	1.36	0.53–3.46	0.521	3.8	1.42–10.14	0.008	2.77	1.26–6.13	0.012
age (15–39 years/> 40 years)	2.58	1.07–6.21	0.035	2.38	0.89–6.41	0.086	2.17	0.97–4.82	0.058
bednet (used/not used)	2.42	0.32–18.51	0.394	1.92	0.25–15.08	0.534	0.77	0.47–1.25	0.284
**Variables at village level : (increasing distances)**									
	**OR**	**95% CI**	**p**	**OR**	**95% CI**	**p**	**OR**	**95% CI**	**p**
distance to forest	1.16	0.81–1.66	0.212	0.84	0.64–1.10	0.103	0.88	0.79–0.97	0.005
distance to health center	0.95	0.77–1.17	0.299	1.04	1.03–1.05	< 0.001	1.00	0.97–1.03	0.435

The multivariate logistic regression model evaluated potential risk factors of *Plasmodium spp*., *Plasmodium falciparum *and *Plasmodium vivax *infections at two levels: gender, age and bednet use at the individual level (interview of surveyed individuals), distances to evergreen forest and to public health centers at the village level (spatial analysis). For the individual level, odds ratios (OR), 95% confidence intervals (95% CI) and *p *values are given for the tested versus the reference value of each variable. For the village level, odds ratios (OR), 95% confidence intervals (95% CI) and *p *values were calculated for increasing distances (in km).

### Risk factors and relative importance of *P. falciparum *and *P. vivax*

There was no major difference between the risk factors for *P. falciparum *and *P. vivax *infections (Table 3). In particular, the association of distance-to-forest and distance-to-health-facility with malaria infection was identical for both species. Individuals aged 15–39 years tended to be at similar risk in all regions and for both species. However, the risk of *P. vivax *in Koh Kong and Preah Vihear was higher for children aged < 15 years and was not associated with gender. In Sampovloun, *P. vivax *infections were more frequent and accounted for more *Plasmodium spp*. infections in < 15 years old children than in individuals ≥ 15 years old (73.3% versus 45.2%, *p *= 0.007). We also observed a higher proportion of *P. vivax *cases in some villages with low malaria prevalence (< 5%) than in the others (53.7% versus 26.4%, *p *< 0,001).

## Discussion

Accurate information on incidence and prevalence is invaluable for planning control activities and monitoring their efficacy over time. It is also an indicator of effectiveness of the methods used for evaluating the impact of malaria on public health and economy [20,21]. Models based on epidemiological and demographic data predict that the actual malaria incidence level is broadly underestimated by routine HIS due to the uneven coverage of the public health facilities and the number of recorded cases treated by the private health sector.

In this study, the first large population-based survey was conducted to provide baseline parasitologic information for populations living in western Cambodia, and to assess the extent to which the malaria situation depicted by the Cambodian HIS is consistent with data collected using an active case detection approach. Unsurprisingly, a much higher prevalence than reported by the national HIS was observed. The discrepancy of the fever-associated *Plasmodium *infections with the time and space-adjusted clinical malaria cases recorded by the HIS showed large geographical variation. Estimated factors of discrepancy between passive and active case detection ranged from 5-fold in Sampovloun to more than 100-fold in Koh Kong. As translation of malaria point prevalence to incidence is not straightforward, these values are only indicative of the size and magnitude of under-reporting through routine HIS. Obviously, this system does not catch a significant proportion of cases and, as a result, downplays certain priorities for malaria control. For example, the low reported annual incidence of malaria cases in Koh Kong per year (API = 5.5 per 1,000 individuals) contrasts with the high prevalence of symptomatic malaria carriers detected in the study population (16 cases per 1,000 individuals during the survey, see Table 2). Better estimates are captured by the HIS at Sampovloun and Preah Vihear with API of 36.4 and 39.2, respectively, as compared to 8.6 and 40 clinical cases per 1,000 detected during the survey in these areas. These findings are likely to reflect the unequal distribution of public health facilities across the country [22] and the strong competition of the private health sector in Cambodia [12,13]. This cross-sectional survey provides a point prevalence of malaria in three provinces bordering Thailand. Additional studies need to be done at different time points during the year and in other areas of Cambodia to better define the major points of divergence with current perception of the malaria situation via HIS.

The risk factor analysis provided a better understanding of local risk and malaria transmission patterns. Obviously, the distance to forest fringe and health facilities have a major impact on malaria transmission in Cambodia. Unsuspected foci, such as the hyperendemic villages in the remote Cardamom mountains of Koh Kong, should be taken into account in future control activities, especially with regards of the marked prevalence of multidrug resistant malaria in these regions [5,14,15]. The increased infection risk associated with increased distance from health facilities points to the need to improve access to health care, especially in remote areas. Village malaria workers (VMW) or outreach activities in such areas can help until the infrastructures improve and population stabilizes. In Koh Kong, and particularly in Preah Vihear, the higher infection risk for children, and the effect of distance to forest and bednet use, are consistent with transmission occurring in forest-fringe villages. Therefore, personal control measures such as impregnated bed-net use should be promoted or reinforced in these villages. In Sampovloun, risk of infection was highest for individuals aged 15–39 years mostly involved in farming and forest activities outside the village, suggesting that infection with malaria parasites occurs frequently in remote forest camps or new settlements and not in long-established villages. Thus, the distribution of impregnated hammock nets and information of forest workers on preventative behavior should be strengthened in this area. Two reasons account for the under-representation of adult men in our study population: i) individuals working outside their villages for farming and forest activities were not present the day of the survey; ii) this age group is poorly represented in Cambodian population which is characterised by a specific deficit of males among the adult population because of excess mortality from civil war [23]. Beside this limitation, this group was at higher risk of malaria in all survey areas, with an age-gender interaction being detected in Sampovloun. In the 15–39 year age group, males are likely to be more exposed to local malaria vectors due to farming or forest activities (woodcutting, hunting, gemstone mining), working with the upper body uncovered and staying outside late at night with no bednet protection [4,24,25]. These observations are consistent with previous studies on forest malaria and malaria risk for men in South-East Asia [24-27]. The spatial distribution of prevalence and the identification of the villages at malaria risk point to a stratified malaria endemicity in Cambodia. Considering the patchy situation of malaria in this country, control strategies should therefore be primarily designed for and adjusted to the village level.

For the first time the rate of fever-associated malaria infections was documented at the community level. A low proportion of fever cases was attributable to malaria, confirming that fever is a poor indicator for presumptive treatment of malaria, even in hypoendemic areas [28]. A large overlooked reservoir of asymptomatic malaria infections was identified in all three regions. This is particularly obvious in Sampovloum area were transmission is low. The conclusion is that the existence of such reservoir of malaria parasites should be considered in the future follow-up of control measures. Longitudinal studies are needed to assess the variation of asymptomatic parasite carriage over time, and its exact contribution to transmission. A complementary approach to continuous monitoring of clinical case at health facilities would be to perform population-based prevalence studies on a regular basis, possibly every second or third year. In this respect, a systematic survey of areas and populations at highest risk such as new settlements, remote rural and forested areas, adult male forest transmigrants, is the priority.

*P. vivax *was responsible for more infections than reported by the HIS [3], accounting for half of malaria cases in Sampovloun. The treatment seeking behavior of Cambodian patients may explain the underestimation of *P. vivax *infections by the HIS [12,13]. *P. vivax *infections do not lead to complications as *P. falciparum *and are usually treated by chloroquine which is readily available in the private health sector. A higher frequency or risk of *P. vivax *infections was observed in children, in contrast to the higher *P. falciparum *infection risk in groups involved in forest activities. The proportion of *P. vivax *infections was also higher in villages with low malaria prevalence and better access to health care facilities, whereas *P. falciparum *dominated in high-risk areas. This may be due to an easy access and use of anti-malarial drugs in villages, especially for children. Indeed, adequate treatment is expected to clear radically *P. falciparum*, whereas *P. vivax *parasites persist as hypnozoites that may relapse. The elimination of *P. falciparum *may even promote reappearance of cryptic *P. vivax *in mixed infections [29-31]. Whether the extensive use of artesunate plus mefloquine combination since 2000 has contributed for the changes observed in the *falciparum *to *vivax *ratio is still uncertain. Interestingly, Ratcliff and Colleagues recently showed higher parasitological failure with *P. vivax *after artemether-lumefantrine than after dihydroartemisinin-piperaquine treatment, as results of the shorter half life of lumefrantrine compared to piperaquine [32]. The mefloquine pharmacokinetic being similar to that of piperaquine, an increasing prevalence of *P. vivax *infections under artesunate plus mefloquine in western Cambodia might therefore indicate a reduced susceptibility of *P. vivax *in this area. This question definitely needs to be further explored. An alternative, but not exclusive explanation, could be higher *P. falciparum *transmission in forests and higher *P. vivax *transmission inside villages. Indeed, malaria and particularly *P. vivax *transmission by minor vectors has been reported in neighbouring countries in villages surrounded by rice fields [33,34]. Compilation of province-based HIS data from 1999 to 2002 indicated that *P. vivax *incidence increased in the north-western Battambang and Pailin provinces despite a decreasing *P. falciparum *incidence. This contrasts with the marked decrease of *P. falciparum *and moderate decrease of *P. vivax *in the rest of the country. A change from dominant *P. falciparum *to dominant *P. vivax *was observed in 1996 on the Thai side of this particular area [11,30,35]. Whether the resurgence of *P. vivax *infections in some areas is linked to specific vectors or topography affecting the distribution of mosquito breeding habitats is not clear and requires further investigation.

In conclusion, this study confirms that the current CNM control strategies such as the distribution of impregnated bednets and the setting-up of VMW in forest-fringe villages are useful, efficient and should be extended [3,4]. At the same time, it raises new questions and recommendations. Data on prevalence and risk factors point to an inadequate description of distribution of species and disease risk in Cambodia, and highlight the need for deployment of additional facilities in under covered areas. Means to capture the cases treated by the private sector should be strengthened [4]. The identification of geographical risk factors will help the mapping of malaria risks, but information on forest coverage should be regularly updated and currently unregistered private health facilities included in the analysis. This, together with ongoing efforts by the CNM to assess the malaria situation better [36], should help reducing the malaria burden in all risk areas of Cambodia

## Authors' contributions

TF and OMP designed the study and were responsible for its coordination. SD and LC were responsible for coordination of the field surveys and the microscopy analysis. SI, LC and PL supervised the surveys and data collection in the field. SN, RS and NK participated in the surveys, the data collection and entry in the database. SI and SV verified and analyzed the data. SI drafted the manuscript, with the help of OMP and TF. All other authors contributed to the data interpretation and critically reviewed the manuscript.
